# Supplementary material for: The interactome of intact mitochondria by cross-linking mass spectrometry provides evidence for coexisting respiratory supercomplexes
Source: Mol Cell Proteomics. 2017 Dec 8;17(2):216–32. doi: 10.1074/mcp.RA117.000470 (PMC5795388; doi:10.1074/mcp.RA117.000470)
Supplement: Supplemental Data [file supp_RA117.000470_133922_0_supp_23978_fzffwf.zip › spectra_annotation/mito_spectra_annotation/207-1-7-1-4-1.pdf]

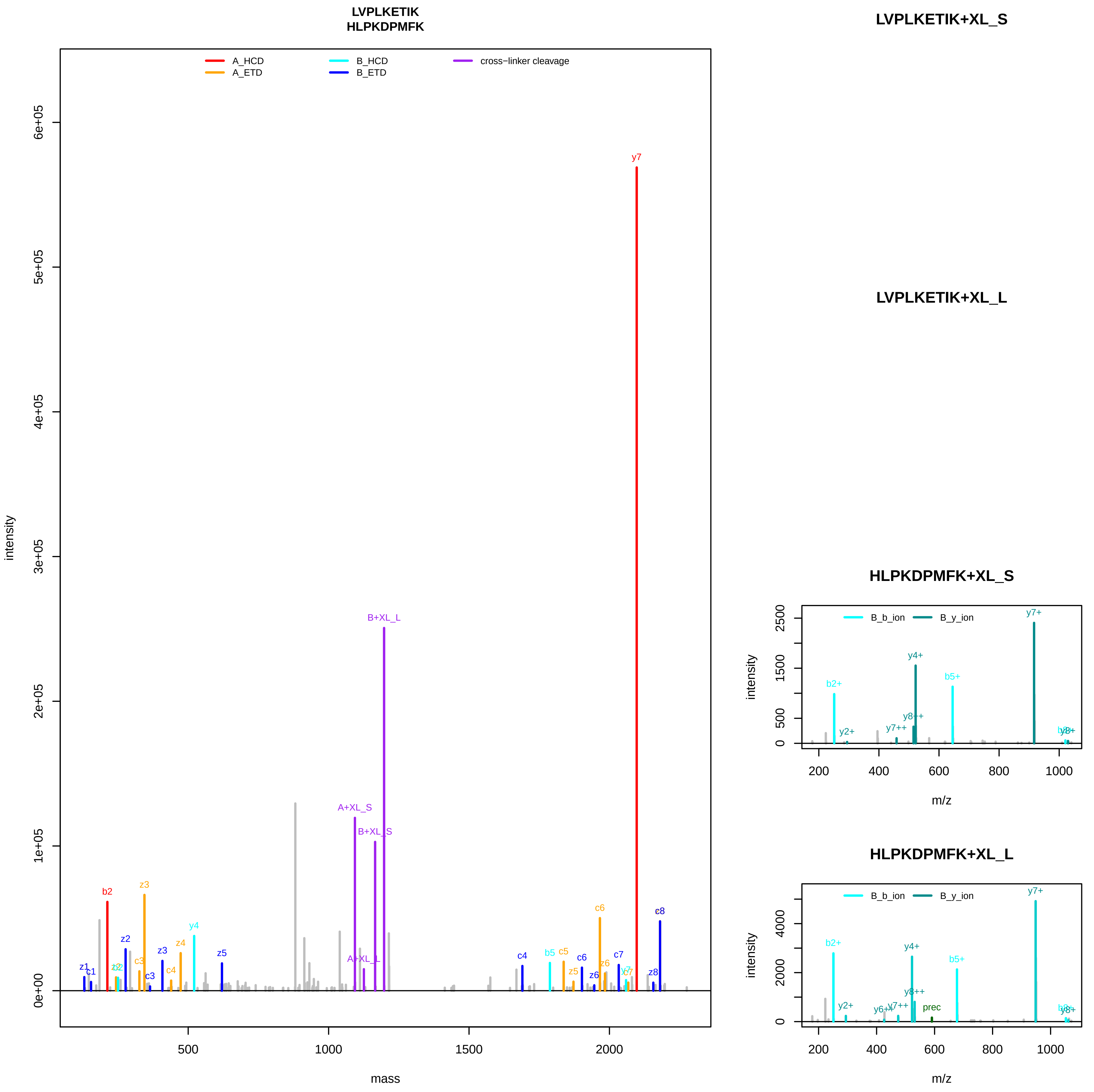
LVPLKETIK
HLPKDPMFK
A_HCD
A_ETD
B_HCD
B_ETD
cross−linker cleavage
intensity
mass
0e+00
1e+05
2e+05
3e+05
4e+05
5e+05
6e+05
500
1000
1500
2000
y7
B+XL_L
A+XL_S
B+XL_S
A+XL_L
b2
z3
z2
z1
c1
c3
z3
c3
c4
z4
y4
z5
c4
b5
c5
z5
c6
c6
z6
z6
c7
c7
z8
c8
LVPLKETIK+XL_S
LVPLKETIK+XL_L
HLPKDPMFK+XL_S
B_b_ion
B_y_ion
y7+
y4+
b5+
b2+
y8++
y7++
y2+
y8+
m/z
0
500
1500
2500
200
400
600
800
1000
HLPKDPMFK+XL_L
y6++
prec
0
2000
4000
